# Use of class IC antiarrhythmic drugs in patients with structural heart disease and implantable cardioverter defibrillator

**DOI:** 10.1007/s00392-024-02394-6

**Published:** 2024-02-19

**Authors:** Maura M. Zylla, Julian Wolfes, Ruben Schleberger, Dennis Lawin, Meinhard Kieser, Florian Reinke, Lars Eckardt, Andreas Rillig, Christoph Stellbrink, Dierk Thomas, Norbert Frey, Patrick Lugenbiel

**Affiliations:** 1 Department of Cardiology, Medical University Hospital, Im Neuenheimer Feld 410, 69120 Heidelberg, Germany; 2 HCR (Heidelberg Center for Heart Rhythm Disorders), Medical University Hospital, Im Neuenheimer Feld 410, 69120 Heidelberg, Germany; 3 DZHK (German Center for Cardiovascular Research), Partner Site Heidelberg/Mannheim, Im Neuenheimer Feld 410, 69120 Heidelberg, Germany; 4 Department of Cardiology II (Electrophysiology), University Hospital Münster, Albert-Schweitzer-Straße 33, 48149 Münster, Germany; 5 Department of Cardiology, University Heart and Vascular Center Hamburg, University Medical Center Hamburg-Eppendorf, Martinistr. 52, 20251 Hamburg, Germany; 6 DZHK (German Center for Cardiovascular Research), partner site Hamburg/Lübeck/Kiel, Martinistr. 52, 20251 Hamburg, Germany; 7 Present Address: Department of Cardiology, Albertinen Heart and Vascular Center, Albertinen Hospital, Süntelstr. 11a, 22457 Hamburg, Germany; 8 Department of Cardiology and Intensive Care Medicine, University Hospital OWL of Bielefeld University, Campus Klinikum Bielefeld, Teutoburger Str. 50, 33604 Bielefeld, Germany; 9 Institute of Medical Biometry, Heidelberg University Hospital, Im Neuenheimer Feld 310, 69120 Heidelberg, Germany

**Keywords:** Class-IC antiarrhythmic drugs, Structural heart disease, Sudden cardiac death, Ventricular arrhythmia, Implantable cardioverter defibrillator

## Abstract

**Background:**

Due to suspected pro-arrhythmic effects and increased mortality associated with class-IC antiarrhythmic drugs (AADs) in previous trials, AAD therapy in structural heart disease (SHD) is mainly restricted to amiodarone. In the presence of diagnostic and therapeutic advancements in cardiovascular medicine, it remains unclear if previous studies adequately reflect contemporary patients. In clinical practice, class-IC-AADs are occasionally used in individual cases, particularly in patients with an implantable cardioverter defibrillator (ICD).

**Methods:**

This study retrospectively investigated outcome in ICD-carriers with SHD in whom class-IC-AADs were used as an individualized therapy due to failure, side effects, or unacceptable risk of alternative therapeutic options.

**Results:**

Fifty patients from four tertiary centers were included (median age 48.5 years; 52% female). The most common underlying SHD were dilated (42%) or ischemic cardiomyopathy (26%) (median LVEF = 45%). Indications for AAD were sustained ventricular arrhythmias (VA) (58%), symptomatic premature ventricular contractions (26%), or atrial arrhythmias (16%). Median follow-up was 27.8 months. Freedom from sustained VA was 72%, and freedom from ICD therapy was 80%. In 19 patients (38%), AAD therapy was terminated. The most common reason was insufficient efficacy (*n* = 8). Pro-arrhythmia was suspected in three patients. Five patients died during follow-up (10.0%), two of cardiovascular cause (4.0%).

**Conclusion:**

In a multicenter cohort of ICD-carriers with SHD, class-IC-AADs were associated with a low rate of pro-arrhythmic effects or cardiovascular mortality. The majority of patients remained free from sustained VA during a follow-up of > 2 years. Further efforts should be made to evaluate the safety of class-IC-AADs in SHD patients receiving contemporary cardiovascular therapy.

**Graphical abstract:**

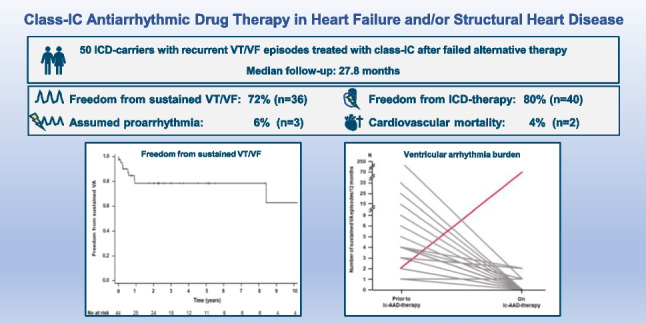

## Introduction

The presence of structural heart disease (SHD) and reduced ejection fraction predispose to ventricular arrhythmias (VA), affecting morbidity and mortality in a relevant subgroup of patients [1]. Pharmacological treatment options are limited as class-IC-AADs have been associated with increased mortality and suspected pro-arrhythmia in patients after myocardial infarction in the “Cardiac Arrhythmia Suppression Trial” (CAST), conducted in the late 1980s [2]. Since CAST, there have been substantial innovations and optimizations regarding cardiac revascularization and heart failure therapy [1]. Importantly, protection from sudden cardiac death (SCD) by implantable cardioverter defibrillators (ICDs) had not yet been established at the time CAST was conducted. Therefore, it remains unclear in how far these data can be transferred to current patient populations and other, non-ischemic cardiac disease entities characterized by different pathophysiological mechanisms. A few recent observational studies have not detected clinically significant pro-arrhythmic effects in arrhythmogenic right ventricular cardiomyopathy (ARVC), left ventricular (LV) hypertrophy, stable coronary artery disease, or children with SHD [3–6]. Nevertheless, chronic medical antiarrhythmic therapy in SHD according to current guidelines is still often restricted to amiodarone [7]. In everyday clinical practice, pharmacological therapy with amiodarone as well as alternative treatment options, e.g., catheter ablation, may be inefficient or carry excessive risk for adverse effects in individual cases [7, 8]. In rare situations, class-IC-AADs are therefore used as a last resort in individual patients after respective informed consent, particularly during in-hospital monitoring or under ICD protection. However, systematic analyses of these cases are sparse.

This study investigates outcome in ICD-carriers with SHD in whom class-IC-AADs were used as an individualized therapy due to failure, side effects, or intolerable risk of other therapeutic options. Due to the limitations in guideline-recommended alternative therapies, systematically analyzing outcome in these cases is of high relevance for everyday clinical practice. As a retrospective registry analysis, this study is associated with relevant limitations as to statistical and structural evaluation of the data and should be interpreted as hypothesis-generating for future large-scale investigations.

## Methods

This study was conducted in adherence to the regulations set forth by the Declaration of Helsinki and approved by the ethics committee of the Medical Faculty of Heidelberg University (registration number: S-737–2021) as well as the local ethics committees of the participating centers.

### Patient selection

Patients were systematically screened at four tertiary care centers based on electronic patient records and included in a retrospective registry. All participating centers possess extensive experience in treatment of heart failure and VA and provide the entire range of specialized medical and interventional therapies. Patient inclusion criteria were age ≥ 18 years, presence of SCD protection by ICD (including cardiac resynchronization therapy or subcutaneous ICD), a left ventricular ejection fraction (LVEF) < 50% and/or SHD, and continuous treatment with class-IC-AAD for a duration of at least seven consecutive days. The inability to provide informed consent constituted an exclusion criterion.

### Data collection

Fifty patients treated at the participating centers between 12/2002 and 07/2022 were included in the study. Demographic and clinical baseline parameters at initiation of class-IC-AAD therapy were extracted from the electronic patient files at the respective center and recorded in the study database. The follow-up period was defined as the entire duration of documented class-IC-AAD administration. Clinical outcome data and prescribed therapy were acquired retrospectively from clinical documentation in the patient file. Medical follow-up was conducted according to routine standards at the respective participating center. Arrhythmia events were extracted from reports of routine ICD interrogations, emergency visits, or hospitalizations. Data on left ventricular ejection fraction (LVEF) were obtained from echocardiography or cardiac magnetic resonance imaging (MRI) reports. In order to assess the potential effects of class-IC-AAD on cardiac inotropy, we compared LVEF before initiation of AAD therapy to LVEF after 6–18 months of class IC-AAD therapy, whenever data regarding this endpoint were available.

Freedom from sustained VA and freedom from ICD therapy due to VA constituted the main endpoints of this study. Changes in VA burden under class-IC-AAD therapy were analyzed in cases with AAD indication due to previous VA and documented number of events before and after therapy initiation.

### Statistical analysis

The patient cohort was described using summary measures of the empirical distribution. Predefined subgroup analyses were performed with respect to baseline parameters and underlying cardiac conditions. Continuous variables are reported as median (with inter-quartile range, Q1 and Q3). The Mann–Whitney U test was applied for between-group comparisons. Dichotomous variables are presented as absolute and relative frequencies and were compared using Barnard’s unconditional exact test. Due to the exploratory character of this analysis, the *P-*values are interpreted in a descriptive sense and no adjustment for multiple testing was applied. *P*-values < 0.05 were denoted as statistically significant. The statistical analysis was performed using SPSS version 28.0.0 and SAS version 9.4.

## Results

### Baseline characteristics

**Fig. 1:**
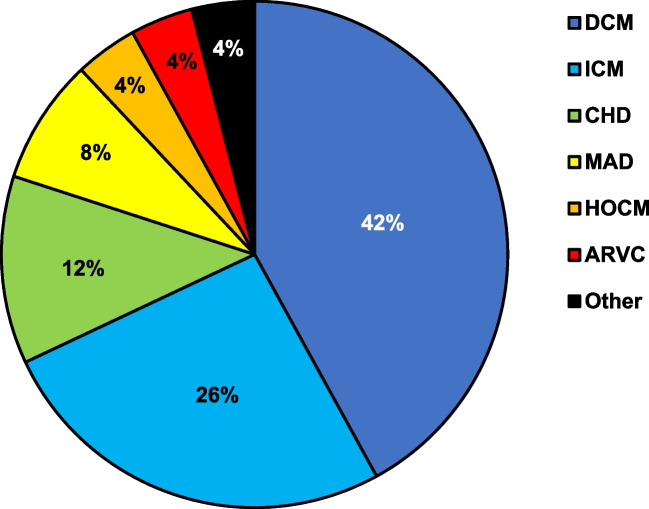
Distribution of underlying cardiac conditions in the study cohort. ARVC, arrhythmogenic right ventricular cardiomyopathy; CHD, congenital heart disease; DCM, dilated cardiomyopathy; HOCM, hypertrophic obstructive cardiomyopathy; ICM, ischemic cardiomyopathy; MAD, mitral annular disjunction

Median age of the 50 patients included was 48.5 years (Q1: 37.5y; Q3: 65.5y). Twenty-six patients were female (Table 1). The underlying SHD in the majority of patients was dilated cardiomyopathy (*N* = 21) or ischemic heart disease (*N* = 13). Other forms of SHD included congenital heart disease (*N* = 6), mitral valve prolapse associated with reduced LVEF (*N* = 4), hypertrophic obstructive cardiomyopathy (*N* = 2), and ARVC (*N* = 2) (Fig. 1). One patient had been diagnosed with cardiac sarcoidosis; in another patient, an arrhythmogenic unclassified cardiomyopathy had been described, characterized by non-ischemic regional hypokinesia of the inferior LV wall. Concomitant coronary artery disease was present in nearly a third of patients (Table 1). Median LVEF was 45.0% (Q1:35.0%; Q3:49.3%) (Table 1). The most common indications for AAD were sustained VA, followed by symptomatic premature ventricular contraction (PVCs) and atrial arrhythmias including atrial fibrillation (Table 1). Flecainide was used in 80.0% (*N* = 40) and propafenone in 20.0% of cases (*N* = 10). The most commonly applied daily dosage of flecainide was 200 mg/24 h (*N* = 21) or 100 mg/24 h (*N* = 15). Individual dosing regimens of propafenone were more heterogenous and ranged from 200 mg/24 h to 900 mg/24 h. The majority of patients received concomitant therapy with betablockers, ACE inhibitors/ARBs/ARNI, and aldosterone antagonists (Table 1). Seven patients received antiarrhythmic co-medication with amiodarone. Amiodarone dosage was documented in six patients of whom four received 200 mg/day and two patients received 400 mg/day.

**Table 1:** Baseline characteristics

Patient characteristic		
Age (years), median [Q1; Q3]	48.5	(37.5; 65.5)
Female sex, *N* (%)	26	(52.0)
Concomitant coronary artery disease, *N* (%)	16	(32.0)
Indication for AAD therapy, *N* (%)		
Sustained ventricular arrhythmia	29	(58.0)
Frequent/symptomatic PVCs	13	(26.0)
Atrial arrhythmia/atrial fibrillation	8	(16.0)
Left ventricular ejection fraction, *N* (%)		
≥ 55%	5	(10.0)
45–54%	21	(42.0)
35–44%	14	(28.0)
≤ 35%	10	(20.0)
Co-medication, *N* (%)		
Betablocker	48	(96.0)
ACE-I/ARB/ARNI	29	(58.0)
Aldosterone antagonist	25	(50.0)
Amiodarone	7	(14.0)

*AAD*, antiarrhythmic drug; *ACE-I*, angiotensin-converting-enzyme–inhibitor; *ARB*, aldosterone-receptor-blocker; AR*NI*, angiotensin receptor/neprilysin inhibitor; *PVC*, premature ventricular complex

### Clinical outcome

**Fig. 2:**
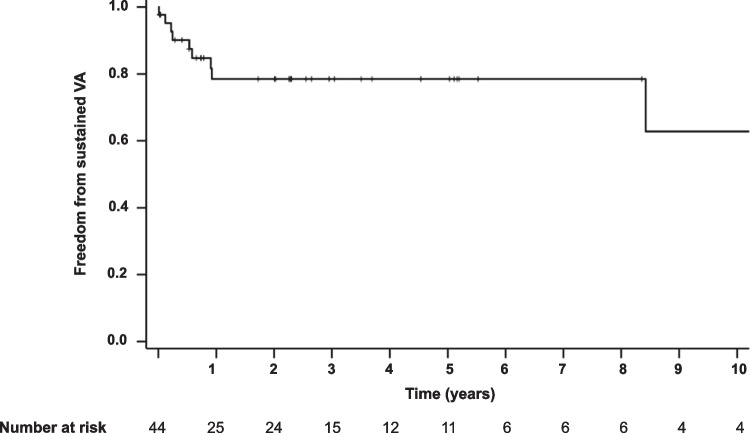
Freedom from ventricular arrhythmia during long-term follow-up. Kaplan–Meier curve depicting freedom from ventricular arrhythmia. Patients with arrhythmia events but without documented time to event were excluded from this analysis. VA, ventricular arrhythmia

**Fig. 3:**
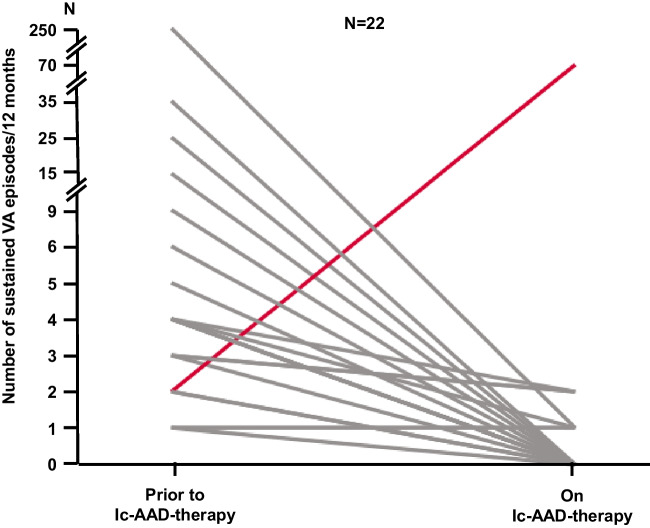
Change in arrhythmia burden under class-IC-AAD. Patients with previous ventricular arrhythmia episodes and documented arrhythmia burden 12 months before and during therapy with class-IC-AAD were included in this analysis (*N* = 22). Grey lines depict patients with a decrease or stable arrhythmia load, and red line shows a patient with an increase in number of ventricular arrhythmias who experienced electrical storm under therapy with flecainide. AAD, antiarrhythmic drug; VA, ventricular arrhythmia

Median follow-up duration under class-IC-AAD was 27.8 months (Q1: 7.2 months; Q3: 62.4 months). During follow-up, 72.0% (*N* = 36) of patients remained free from sustained VA (Fig. 2), and 80% (*N* = 40) of patients were free from ICD therapy. In a subgroup of 22 patients with indication for AAD due to sustained VA, clinical documentation regarding the number of arrhythmia events during 12 months prior to initiation of AAD therapy was available. Comparing VA burden 1 year before and 1 year after initiation of class-IC medication in these patients, a statistically significant reduction in sustained arrhythmic events under class-IC-AAD could be observed (before: median 4 events/year [Q1: 2.0 events/year; Q3: 6.8 events/year]; after: median 0.0 events/year [Q1: 0.0 events/year; Q3: 1.0 event/12 year], *P* = 0.001). One male patient with ischemic cardiomyopathy and severely reduced LVEF experienced an electrical storm under class-IC-AAD. In two patients with one previous episode of VA, another single episode was observed during the time of AAD therapy. In all other patients, a reduction in VA burden could be observed (Fig. 3).

**Fig. 4:**
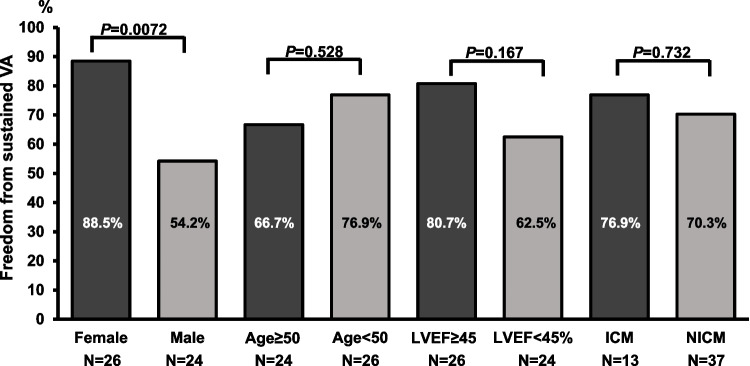
Freedom from sustained ventricular arrhythmia in patient subgroups. Rates of freedom from ventricular arrhythmia in demographic and clinical subgroups. ICM, ischemic cardiomyopathy; LVEF, left ventricular ejection fraction; NICM, non-ischemic cardiomyopathy; VA, ventricular arrhythmia

Women were more often free from VA events than men (Fig. 4). Age ≥ 50 years, LVEF < 45%, or underlying ischemic cardiomyopathy were not associated with a higher incidence of VA recurrence under class-IC-AAD (Fig. 4). Presence of coronary artery disease either as a primary cardiac condition or co-morbidity was also not associated with a higher rate of VA (*P* = 0.804). The difference in VA recurrence between patients with (*N* = 7) or without co-medication with amiodarone (*N* = 42) was not statistically significant (amiodarone + class-IC-AAD: 57.1%; class-IC-AAD without amiodarone: 23.3%, *P* = 0.069).

**Fig. 5:**
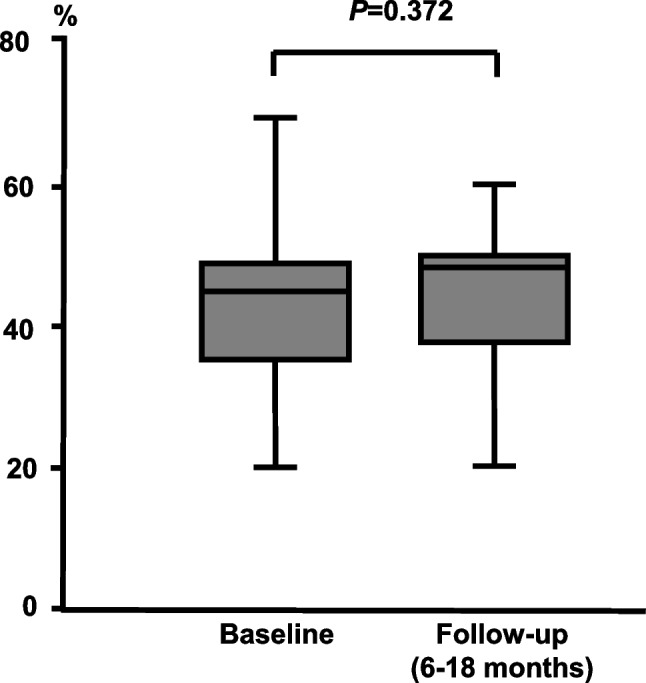
Left ventricular systolic function before and under class-IC-AAD. Left ventricular ejection fraction is shown before initiation of AAD therapy and during follow-up on the respective therapy in patients with documented echocardiographic or MRI-based measurements both at baseline and during follow-up (*N* = 25)

With respect to potential negative inotropic effects of class-IC-AAD, there was no statistically significant change in LVEF in patients with a documented LVEF at baseline and follow-up at 6–18 months under AAD therapy (*N* = 25, Fig. 5).

Five patients died during follow-up (10.0%). Two patients died of cardiovascular cause; one patient died due to a non-cardiovascular condition. In two other cases, the cause of death was unknown.

In 19 patients (40%), class-IC-AAD therapy was terminated during follow-up. The most common reason for termination was perceived insufficient antiarrhythmic efficacy (*N* = 8). Of these patients, one patient experienced multiple ventricular arrhythmias during follow-up which were attributed to a progression of the underlying ischemic cardiomyopathy rather than pro-arrhythmic effects of AAD therapy; one patient actually showed a reduction in VA episodes on re-evaluation of the ICD-interrogation; three patients had received class-IC-AAD due to atrial arrhythmias. In the remaining three patients of this subgroup, information on arrhythmia burden before and after AAD was incomplete. Pro-arrhythmia was suspected in 3 patients (6%) according to assessment by the prescribing physician. These patients experienced multiple recurrent VA events under class-IC-AAD, adequately terminated by ICD therapy. Two of these cases had received class-IC-AAD due to previous sustained VA events, and underlying cardiac diseases were ischemic cardiomyopathy (LVEF = 50%) and dilated cardiomyopathy (LVEF = 40%), respectively. In the third case, AAD had been prescribed due to atrial arrhythmia in congenital heart disease (transposition of great arteries, LVEF = 40%). In the subgroup of patients treated with class-IC-AAD for atrial arrhythmias (*N* = 8), this was the only patient in whom VA events and potential pro-arrhythmia were observed, leading to discontinuation of AAD therapy. In the patient with ischemic cardiomyopathy, an increase in QRS duration had been observed under class-IC-AAD (from 168 to 200 ms under QRT stimulation). In the other two patients in whom class-IC-AADs were discontinued due to suspected pro-arrhythmic effects, QRS duration remained unchanged during AAD therapy (90 and 100 ms, respectively).

Eleven patients received additional catheter ablation of VA during follow-up (22.0%); in five of these cases, AAD therapy was discontinued after CA.

## Discussion

This study evaluates rhythm-associated and prognostic outcomes in a multicenter cohort of selected patients receiving class-IC-AAD therapy despite the presence of SHD and, thus, outside of guideline recommendations. In all patients, SCD-protection and arrhythmia monitoring was established with previously implanted ICD systems. We show a low rate of recurrent VA events and cardiovascular mortality with class-IC-AAD therapy in this selected cohort. In patients with previous VA events, a reduction in arrhythmia burden in comparison to baseline could be observed.

Class-IC-AADs attenuate arrhythmogenicity by binding to atrial and ventricular sodium channels and increasing excitation threshold, slowing intracardiac conduction and reducing overall excitability [9]. Clinical applications according to current guidelines include the treatment of supraventricular or ventricular arrhythmias in the absence of structural heart disease [7]. CAST examined the effect of class-IC-AADs on ventricular ectopy and sudden cardiac death in post-myocardial infarction patients. The trial was prematurely terminated because of an excess of deaths due to arrhythmia or shock after acute myocardial infarction in patients treated with encainide or flecainide [2]. As a consequence, current guidelines restrict the use of class-IC-AAD in SHD. Even though the CAST cohort consisted of survivors of myocardial infarction, the conclusions of the trial were generalized to the entire and heterogenous population of patients with SHD and/or heart failure [7, 10]. Nevertheless, data on the safety of class-IC-AAD in important subgroups, e.g., heart failure due to non-ischemic cardiomyopathy or SHD without ischemic substrate or scars, are missing. Additionally, an unequivocal arrhythmia-related increase in mortality in CAST can be debated as relevant nonlethal proarrhythmogenic effects could not be observed during the ECG-monitored dose titration phase [2]. Any sudden, unexplained case of death during follow-up was classified as arrhythmia-related death according to the CAST protocol [2]. Furthermore, several limitations to CAST have to be acknowledged in the light of current treatment strategies. The CAST trial was conducted in 1987–1989. In the last decades, there have been substantial advancements in heart failure therapy and cardiac revascularization, as well as in SCD prevention by ICD implantation for primary and secondary prevention indications.

In stable coronary artery disease, retrospective observational studies have shown a low rate of adverse clinical events under class-IC-AAD and indicated a beneficial outcome compared to class-III-AAD [5, 11]. Additionally, observational studies on patients with LV hypertrophy or ARVC have produced encouraging results regarding the safety and efficacy of class-IC-AAD in other forms of SHD [3, 4]. Nevertheless, current guideline-adherent therapy restricts antiarrhythmic therapy in patients with SHD to amiodarone which is associated with multiple severe adverse effects, frequently necessitating discontinuation of this therapy [7].

On the other hand, amiodarone is often used as first-line therapy in young patients without coronary artery disease or SHD despite eligibility for alternative, better-tolerated AAD options [12, 13]. This reflects a potential insecurity of prescribing physicians with respect to the safety profile of class-IC-AAD, especially in centers less experienced in specific arrhythmia therapy or in patients with unknown cardiac co-morbidity [13].

Our study adds to the current evidence of class-IC-AAD applications beyond current guidelines in a cohort of ICD-carriers with heart failure or other forms of SHD, contraindication for amiodarone, or lack of other therapy options. Even though this selected cohort included patients with SHD of different etiology, all patients were characterized by a previous indication for ICD protection for primary or secondary prevention and, thus, a high risk for VA. Continuous rhythm monitoring by ICD over a prolonged median follow-up period constitutes an additional strength of this study, enabling detection of potential pro-arrhythmic effects.

In this cohort, class-IC-AAD therapy was associated with a reduction in arrhythmia burden and a low rate of VA recurrence, even despite substantial baseline risk for VA. The majority of patients in our cohort received modern heart failure medication, in particular betablocker therapy, which had been present in only a quarter of patients in the CAST cohort. Thus, optimizing heart failure therapy in addition to antiarrhythmic medication may be one strategy to achieve an overall favorable clinical outcome. However, this has to be confirmed in future large-scale, prospective studies.

Most patients in this cohort were characterized by a moderately reduced LVEF. In CAST, there was no difference in rates of supposedly arrhythmogenic cardiac arrest between patients with LVEF < 30% or ≥ 30%. In our subgroup analyses, neither LVEF < 45%, ischemic cardiomyopathy as an underlying cardiac condition, nor the presence of coronary artery disease were associated with an elevated rate of VA recurrence. Female patients showed reduced VA recurrence in comparison to male patients, indicating a sex-associated effect. Sex-specific differences in flecainide metabolism leading to higher clearance and lower serum concentrations in males have been described and may contribute to this observation [14]. However, in previous studies evaluating sex-specific outcomes under both class-I- and class-III-AADs, female patients experienced more adverse drug-related effects [15]. Additionally, sex-related differences in antiarrhythmic substrate may play a role [16]. Whereas the number of male and female patients was balanced in our cohort, female patients are underrepresented in trials on VA therapy. Sex-specific pathophysiology and rhythm-associated outcome under class-IC-AAD should be further investigated by future large-scale trials.

Negative inotropic effects of flecainide have been proposed as additional caveats for its administration in the context of heart failure [2, 17]. In a subgroup of patients with documented LVEF at follow-up under class-IC therapy, we did not detect a significant decrease in systolic LV function. This may constitute a reassuring signal for therapeutic safety with respect to hemodynamic effects. However, as median LVEF at baseline was only moderately reduced in this cohort, we cannot preclude relevant effects in patients with severely reduced systolic function. Therefore, any antiarrhythmic therapy should be implemented under regular clinical and echocardiographic follow-up in patients with heart failure.

A small subgroup of patients received co-medication with amiodarone as part of the individualized antiarrhythmic therapy. As the subgroup analysis provided in this study includes only a few patients, no reliable conclusion with respect to the safety of this co-medication can be made. In clinical practice, combination therapy of different antiarrhythmic agents should be performed with caution and under close monitoring due to potentially critical side effects.

In only a minority of patients (*N* = 3), AAD therapy was terminated due to proposed proarrhythmic effects according to assessment by the prescribing physician. Unfortunately, the VA burden before initiation of class-IC-AAD was not documented in two of these cases. In the third case, only atrial arrhythmia was documented during 12 months prior to AAD therapy. These three cases were each diagnosed with different underlying cardiac conditions and LVEF ranging from 40 to 50%. Predictors for potential pro-arrhythmia under class-IC-AAD cannot be reliably identified based on these data. However, these data show that possibly patient-specific or substrate-specific characteristics rather than the underlying SHD itself may play a role. The most common reason for termination of AAD therapy was perceived insufficient efficacy in individual cases. Accordingly, AADs were discontinued after catheter ablation of VA in a relevant number of cases. Unfortunately, without a control group, confirming these notions of reduced efficacy is not possible. However, comparison of arrhythmia burden before and after initiation of AAD and overall rates of VA recurrence in this cohort rather indicate a beneficial rhythm-associated outcome in the majority of patients. Large-scale trials are needed to investigate individual predictors both for the safety and efficacy of class-IC-AAD in SHD for an optimized stratification and personalization of antiarrhythmic therapy.

### Limitations

Due to its retrospective, observational, single-cohort design, the study carries inherent limitations. The cohort size is limited, particularly regarding the subgroup analyses, and the heterogeneity of underlying SHD entities constitutes an additional limitation regarding disease-specific evaluation of the results. However, cases receiving class-IC-AAD outside of current guideline recommendations in real-world clinical practice constitute a highly selected patient clientele. The prerequisite of ICD protection additionally contributed to this rigorous pre-selection but, on the other hand, enabled analyzing data from continuous rhythm monitoring documented in the patient file and device interrogation reports. However, generalization of the observations to patients without ICD protection is not possible. Clinical follow-up intervals, duration, and associated diagnostic procedures differed between centers and individual patients. Thus, data on LVEF under AAD therapy or arrhythmia burden before therapy initiation were not available for every patient at standardized time points. The respective number of patients with available data is indicated in the text or shown in the respective tables and figures, whenever information was available only for a subgroup. Additionally, data on PVC burden under class-IC-AAD in these patients would offer additional insight into the pro- or antiarrhythmic potential of this therapy in SHD. However, PVC burden was not systematically documented in the respective patient files and could therefore not be evaluated in this study. In light of these limitations, this study has to be interpreted as hypothesis-generating. However, we believe that these data contribute to paving the way for future, larger-scale clinical trials re-evaluating the efficacy and safety of class-IC-AAD in heart failure and SHD under modern cardiovascular therapy standards.

### Conclusion

In a multicenter cohort of patients with SHD at high risk for VA, we show a low rate of recurrent VA events and cardiovascular mortality under individualized class-IC-AAD therapy during a prolonged median follow-up period of 27.8 months. Female patients were characterized by lower rates of VA recurrence than male patients. No significant adverse effects on systolic LV function were detected in this cohort. This study may support new larger-scale, prospective trials to re-evaluate the use of class-IC-AAD in SHD patients. Furthermore, patient- or substrate-specific predictors for outcome under class-IC-AAD, beyond the “traditional” indicators of LVEF and underlying heart disease, may be of additional importance and should be the target of future scientific endeavors.

## Data Availability

The underlying data of this study are available from the corresponding author upon reasonable request and as far as compatible with personal data protection regulations.
